# Electrically Controllable Microparticle Synthesis and Digital Microfluidic Manipulation by Electric-Field-Induced Droplet Dispensing into Immiscible Fluids

**DOI:** 10.1038/srep31901

**Published:** 2016-08-18

**Authors:** Taewoong Um, Jiwoo Hong, Do Jin Im, Sang Joon Lee, In Seok Kang

**Affiliations:** 1 Department of Chemical Engineering, Pohang University of Science and Technology (POSTECH), San 31, Hyoja-Dong, Nam-Gu, Pohang, Gyeongbuk, 37673, South Korea; 2 Department of Mechanical Engineering, Pohang University of Science and Technology (POSTECH), San 31, Hyoja-Dong, Nam-Gu, Pohang, Gyeongbuk, 37673, South Korea; 3 Department of Chemical Engineering, Pukyong National University, 365 Sinseon-ro, Nam-gu, Busan 48547, South Korea

## Abstract

The dispensing of tiny droplets is a basic and crucial process in a myriad of applications, such as DNA/protein microarray, cell cultures, chemical synthesis of microparticles, and digital microfluidics. This work systematically demonstrates droplet dispensing into immiscible fluids through electric charge concentration (ECC) method. It exhibits three main modes (i.e., attaching, uniform, and bursting modes) as a function of flow rates, applied voltages, and gap distances between the nozzle and the oil surface. Through a conventional nozzle with diameter of a few millimeters, charged droplets with volumes ranging from a few μL to a few tens of nL can be uniformly dispensed into the oil chamber without reduction in nozzle size. Based on the features of the proposed method (e.g., formation of droplets with controllable polarity and amount of electric charge in water and oil system), a simple and straightforward method is developed for microparticle synthesis, including preparation of colloidosomes and fabrication of Janus microparticles with anisotropic internal structures. Finally, a combined system consisting of ECC-induced droplet dispensing and electrophoresis of charged droplet (ECD)-driven manipulation systems is constructed. This integrated platform will provide increased utility and flexibility in microfluidic applications because a charged droplet can be delivered toward the intended position by programmable electric control.

The dispensing of tiny droplets containing chemical or biological materials has gained increased interest for several practical applications, such as biological microarrays^1^,^2^, vesicle formation^3^,^4^, micro/nanoparticle synthesis^5^,^6^, and digital microfluidics^7^,^8^. Various methods for droplet formation, including laser-guided printing^9^, droplet-based microfluidics^10^,^11^, inkjet jetting^12^, and electrohydrodynamic (EHD) jetting^13^, have been proposed to develop such applications. Highly detailed information on droplet formation techniques is provided in reviews^14^,^15^ and references.

In particular, the EHD jetting method, which is atomization of a liquid by an applied electric field between the nozzle and the bottom electrode, has attracted significant attention because it can generate droplets with sizes ranging from millimeters to micrometers without reducing the size of a capillary nozzle^16^,^17^. This method can resolve the technical problems of conventional inkjet printing, such as clogging from the use of chemical or biological samples and consequential damage of inkjet nozzle^16^,^17^. On the basis of these advantages, the EHD jetting method has been successfully utilized in numerous applications, including printed electronics, DNA/protein arrays, and self-assembly of nanomaterials^5^,^6^. However, given the superiority and the practical applicability of the EHD jetting method, it still poses technical problems, like the requirement of a conducting target surface (or electrode) and a highly applied electric potential (>10 kV)^18^,^19^.

The electric charge concentration (ECC) method has been proposed to address these problems. This method can dispense droplets by using the electric attraction between charges on the droplet surface and the induced counter charges on the surface of the target substrate, when the electric potential is applied to the conducting nozzle^18^,^19^. Therefore, droplet formation through ECC method can be utilized regardless of the physical properties of the target substrates (e.g., electric conductivity, electric permittivity, as well as phase) because electric induction occurs in any material. In our previous work, we presented the preliminary results on dispensing picoliter droplets through ECC method on dielectric substrates such as a glass plate and oil^18^,^19^. However, detailed studies on dispensing as influenced by physical factors (e.g., flow rate, applied voltage, and gap distance between the nozzle and the oil surface) and its potential application, have yet to be reported.

This paper presents the first study to systematically investigate the droplet dispensing into oil by ECC method (Fig. 1) and the applicability of the proposed dispensing method to various applications, including synthesis of microparticles and digital microfluidics. The ECC method can generate tiny droplets through the electric attraction between charges on water droplet surface and the induced counter charges on the oil surface (Fig. 1b). The dispensing process will be described in detail later. The dispensing behavior is systematically studied under a wide range of flow rates, applied voltages, and gap distances between the nozzle and the oil surface. The operation modes are characterized by the dispensing behaviors and an empirical relationship between droplet volume, applied voltage, gap, and flow rate is established. The fundamental feature of the proposed system is that electrically charged droplets can be uniformly generated and the amount of the electric charge can be controlled. The electrical polarity of the dispensed droplet also can be altered with the polarity of electric potential applied on the nozzle. Another feature of the proposed method is the direct dispersal of droplets into oil. In this way, the charge relaxation time (~few seconds) of oils which are leaky dielectric with low conductivity is sufficient to maintain the electric charge of the dispensed droplet for an extended period of time. Furthermore, a myriad of applications, including micro/nanoparticle synthesis, are frequently performed in two-phase systems of immiscible liquids such as water–oil systems. On the basis of the aforementioned features, the feasibility of the chemical synthesis of microparticles, such as colloidosomes and anisotropic Janus particles, is ascertained. Finally, the ECC-induced droplet dispensing and manipulation systems are integrated by adopting electrophoresis of charged droplet (ECD), which can provide increased utility and flexibility in digital microfluidics (i.e., microfluidic technology to electrically manipulate discrete droplets on an array of electrodes) and their practical applications.

## Results

### ECC-induced droplet dispensing

**Figure 2:**
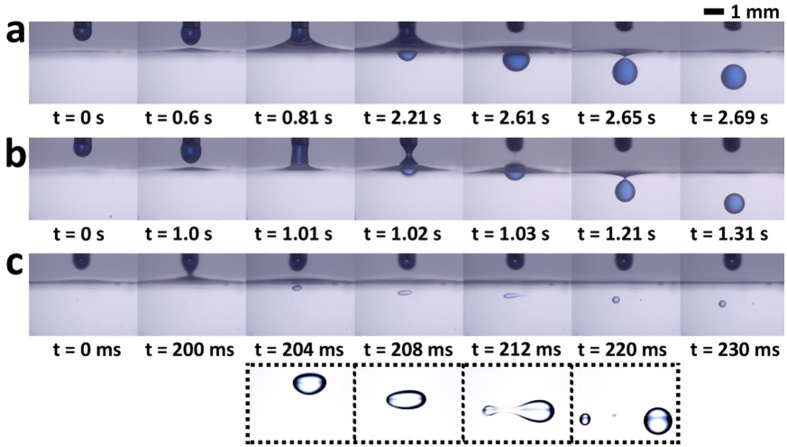
Consecutive side-view images of dispensing droplets with different applied voltages. Droplet dispensing through ECC method exhibits three main modes: (**a**) attaching, (**b**) uniform, and (**c**) bursting modes. The dashed box indicates a close-up of the bursting droplet that breaks up into two smaller droplets beyond the Rayleigh’s charge limit.

The dispensing behavior of droplets induced by ECC method is consecutively observed when different voltages are increasingly applied to the nozzle under fixed flow rate and fixed gap distance conditions (Fig. 2). ECC-induced droplet dispensing exhibits three main modes (i.e., attaching, uniform, and bursting modes) with varying applied voltages, flow rates, and gaps. The three dispensing modes notably exhibit similar dynamic behavior. When an electric voltage is applied to the nozzle, the pendant droplet is elongated toward the oil surface. This surface simultaneously rises toward the pendant drop because of the electrostatic attraction between the charges on the pendant droplet and the induced charges on the oil surface. Subsequently, the pendant droplet and the oil surface touch and form a liquid bridge. Once the liquid bridge is broken by capillary instability, a small droplet is dispensed into the oil chamber.

The dispensing modes are distinguished and defined by formation and break-up of liquid bridge and the uniformity of droplet size. When a relatively small voltage is applied, the deformation is insufficient to break up the liquid bridge right after the contact of the pendant droplet and oil surface. Afterward, oil attaches to the pendant droplet and rises toward the nozzle, which leads to the extension of time duration between the formation and break-up of liquid bridge (>1 s). Accordingly, the size of the dispensed droplet becomes larger. This dispensing behavior is called “attaching mode” because the oil surface appears to attach toward the tip of the nozzle (Fig. 2a). The charge amount of a pendant droplet increases with the applied voltage (Figure S2 in Supplementary Information); thus, the bottom apex of the droplet becomes tapered^20^. The droplet and the oil surface then come into contact with a narrower area. Consequently, the break-up occurs right after formation of the liquid bridge (~30 ms), and the size of the dispensed droplet decreases (Fig. 2b). This dispensing behavior is called “uniform mode” because the droplets are dispensed uniformly in terms of volume and frequency in this mode. When the voltage becomes too high, the dispensed droplet breaks up into smaller droplets in the oil medium because of the Rayleigh’s charge limit (i.e., maximum amount of charge a liquid droplet can carry) (Fig. 2c)^21^,^22^. This dispensing behavior is called “bursting mode”, which is similar to the electrospray mode in EHD jetting^22^. Uniform droplets cannot be obtained at bursting mode because of electric instability.

**Figure 3:**
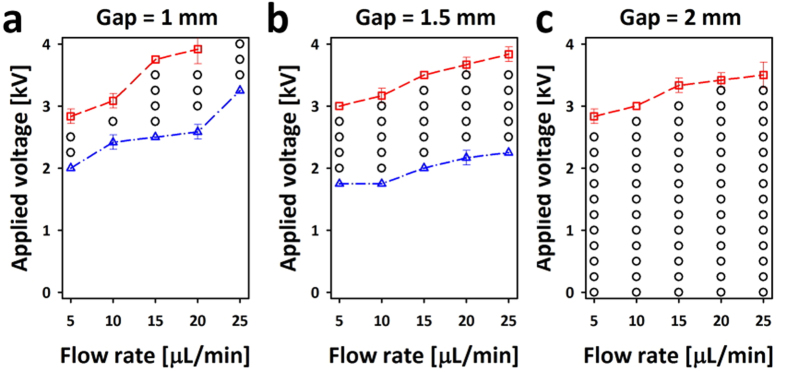
Phase diagram of the operation modes as a function of flow rate and applied voltage at different gap distances between the nozzle and the oil surface of (**a**) 1, (**b**) 1.5, and (**c**) 2.0 mm. Note that the data points were measured by increasing voltage from 0 V at 0.25 kV increments until the busting mode first appeared.

To analyze the dispensing modes quantitatively, the influences of applied voltage, flow rate, and gap distance between the nozzle and the oil surface on the operation modes are investigated (Fig. 3). As the gap between the nozzle and oil surface increases, the attaching mode recedes, and the uniform and bursting modes become dominant. By contrast, the attaching mode becomes dominant and the bursting mode recedes as the flow rate increases. These results provide useful information to find the optimal conditions for the uniform dispensing of charged droplets.

**Figure 4:**
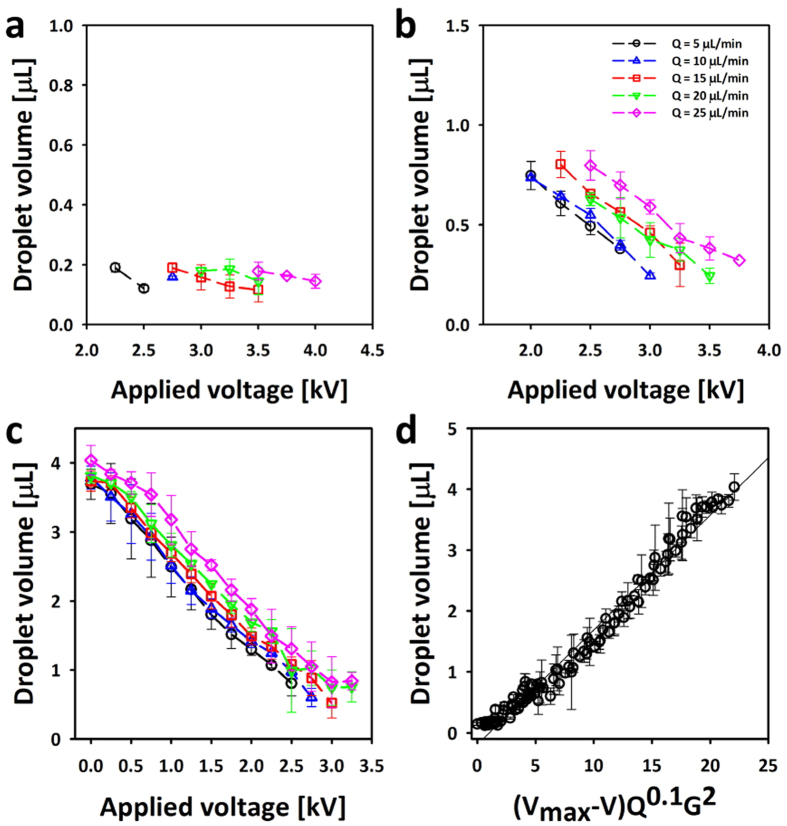
Relationship between droplet volume and parameters. (**a**) gap = 1 mm (**b**) gap = 1.5 mm (**c**) gap = 2 mm. (**d**) Scaling analysis for establishing an empirical relationship between droplet volume, applied voltage (V), gap (G) and flow rate (Q). Note that V_max_ is 4 kV.

Based on a parametric study for dispensing, we found that droplet volume decreases with increasing applied voltage and decreasing gap distance in uniform mode. (Fig. 4). A slight increase in droplet volume as the flow rate varies from 5 to 25 μL/min is observed. Droplets with volumes ranging from 4 μL to 92 nL can be uniformly dispensed into oil chamber without reductions in nozzle size by using a conventional nozzle (inner diameter of 0.6 mm and outer diameter of 0.9 mm).

### Preparation of colloidosomes by electrostatic self-assembly

**Figure 5:**
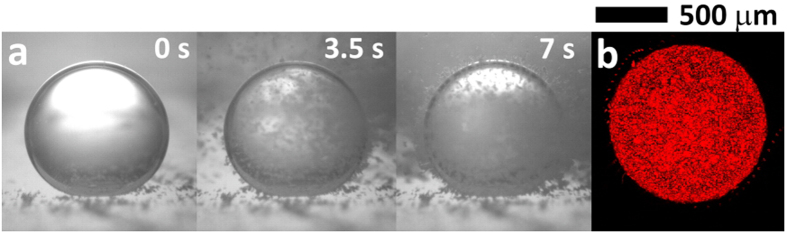
Electrostatic self-assembly of particles onto the charged droplets. (**a**) Time-lapse photographs of the electrostatic self-assembly procedure of PS particles with diameter of 10 μm. Note that the droplet is gently positioned on the PDMS block. (**b**) Confocal image of thick layer of PS-COOH particles with diameter of 2 μm formed on a charged droplet.

To develop an alternative and technologically simple strategy for preparing colloidosomes without any pretreatment process, we examined the self-assembly of colloidal particles onto the interface of charged droplets generated by the proposed ECC method (Fig. 5). The temporal evolution of the self-assembly process is difficult to observe because of its short overall time. Thus, a charged droplet is preferentially dispensed into oil medium and polystyrene (PS) particles are poured into the oil chamber to clearly visualize the self-assembly process. Figure 5a illustrates the dielectrophoretic (DEP) motion of the dispersed particles toward the charged droplet. The polystyrene (the relative permittivity *ε*_*p*_ = 2.4)^23^ and the n-dodecane (the relative permittivity *ε*_*m*_ = 2.0)^23^ are used as microparticles and oil medium in the present experiment, respectively. Here, the DEP force acting on the particles can be calculated by 2*πa*^3^*ε*_*m*_*ε*_0_*f*_*CM*_∇||*E*||^2^, where *a* is the radius of particles, *f*_*CM*_ = (*ε*_*p*_ − *ε*_*m*_)/(*ε*_*p*_ + 2*ε*_*m*_) is Clausius-Mossotti factor for a DC electric potential case and *ε*_0_ is permittivity of free space. Therefore, the direction and the magnitude of dielectrophoretic force on the particles are determined by both *f*_CM_ and ∇||*E*||^2^. When *f*_CM_ becomes positive, a positive DEP force is exerted on the particles, which means that the direction of force is toward where the electric field is stronger. Consequently, the particles start to attach on the charged droplet by the positive DEP force. After the packing of one layer, particles are additionally attached to this layer in a radial manner. This additional attachment may be attributed to the fact that the polarized particles are aligned and aggregated along the electric field line emanated from the charged droplet^23^. In addition, carboxylate-modified PS particles (PS-COOH particles) with a negative surface charge are employed to intensify the electrical attraction of particles onto the positively charged droplet (Figure S4 in Supplementary Information). We ascertain that the surface of the charged droplet is covered by PS-COOH particles by using a confocal laser scanning microscope (Fig. 5b). We believe that the coulomb attraction between the surface of droplet and particles with DEP force will enable the preparation of colloidosomes with greater ease than the conventional methods based on the stabilization of Pickering emulsion^24^. We believe that our method will be a good alternative or complementary technology to fabricate the self-assembly of colloidal nanoparticles^25^,^26^.

### Precipitate formation by coalescence of oppositely charged droplets

**Figure 6:**
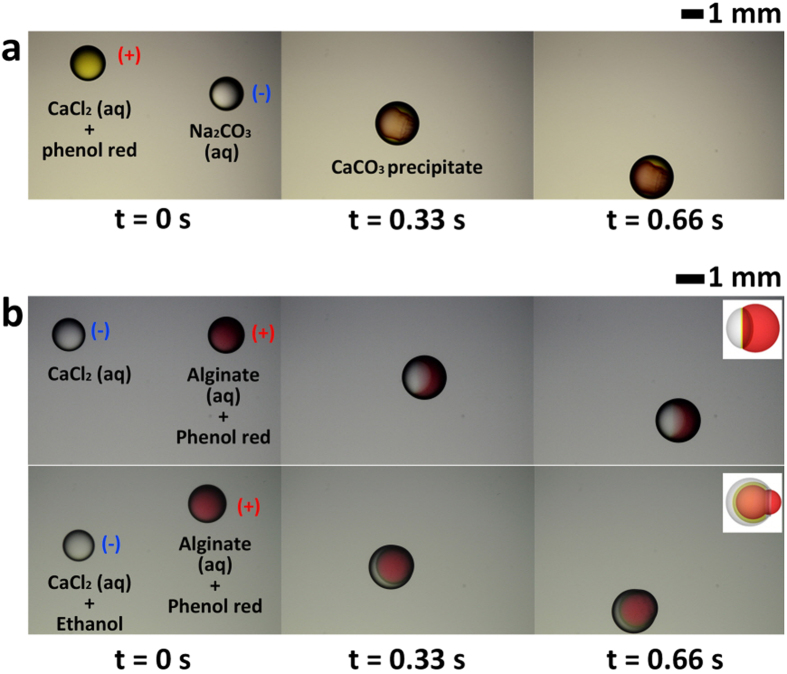
(**a**) Chemical reaction for precipitate formation of CaCO_3_ by coalescence of oppositely charged droplets of CaCl_2_ and Na_2_CO_3_. (**b**) Formation of Janus droplet by coalescence of oppositely charged droplets of CaCl_2_ and SA. The top and bottom rows illustrate formation of Janus droplet with different shapes by the variation in surface tension.

To demonstrate the applicability of the proposed ECC-driven dispensing system for chemical synthesis, we examined the coalescence of oppositely charged droplets containing different chemical samples that produce precipitates through chemical reaction (Fig. 6a). When colorless aqueous solutions of sodium carbonate solution (Na_2_CO_3_) and calcium chloride solution (CaCl_2_) are mixed, a white powdery calcium carbonate (CaCO_3_) precipitate is produced^27^. To observe the formation of CaCO_3_ precipitate distinctly, phenol red is added to the CaCl_2_ solution. Phenol red is used as a pH indicator, which displays red color in the pH range of 6.4 to 8.2^27^. To achieve the precipitate reaction, a positively charged droplet containing CaCl_2_ with phenol red and a negatively charged droplet containing Na_2_CO_3_ are dispensed by applying an equal and opposite voltage to each nozzle. The CaCO_3_ precipitate is subsequently formed through coalescence of the oppositely charged droplets because of electric attraction^28^,^29^. When the two liquids are mixed inside the merged droplet, the color changes to red as the pH value of the mixed solutions becomes 7.7^27^. This simple coalescence method for the precipitate reaction will be primarily directed toward the design and synthesis of nanocrystals with controlled sizes and shapes (or facets) for catalytic applications^30^,^31^. Similarly, calcium alginate is formed near the interface through the coalescence of oppositely charged droplets containing sodium alginate (SA) and CaCl_2_, as shown in the top row of Fig. 6b^32^,^33^. Phenol red is also added to the SA solution to observe the formation of calcium alginate. The formation of calcium alginate is initially detected by the fact that the interface is maintained right after coalescence even though the two liquids are miscible. Afterward, the color changes gradually from red to yellow at the interface of the red droplet containing SA solution, which is slightly alkaline (pH = 8.3), and the transparent droplet containing CaCl_2_ (pH = 5.2) after coalescence, which means calcium alginate is formed near the interface. In addition, Janus droplet with different internal structures can be formed by decreasing the surface tension of the droplet containing CaCl_2_^34^, as shown in the bottom row of Fig. 6b.

### Fabrication of anisotropic Janus microparticles with different shapes

**Figure 7:**
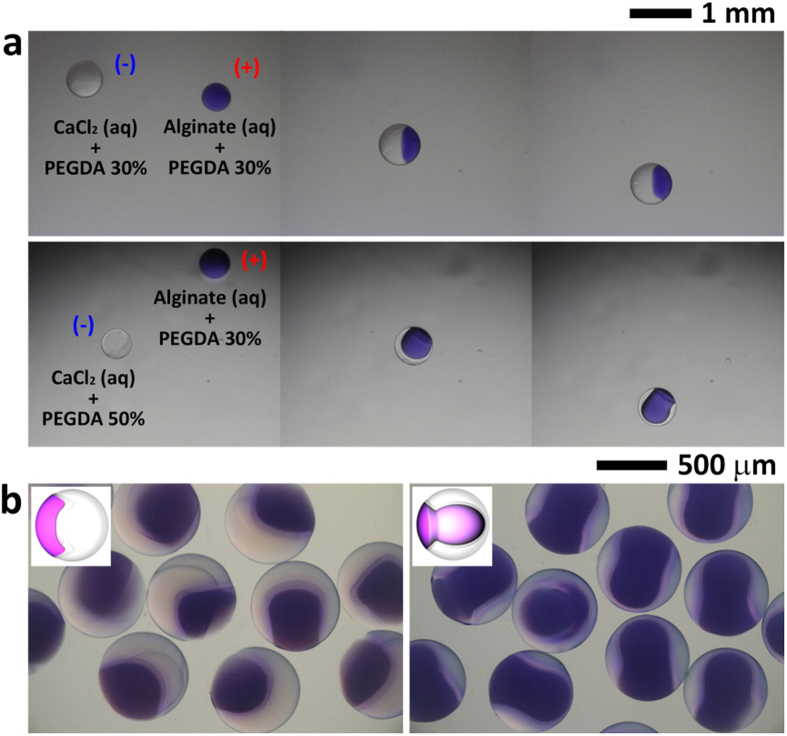
(**a**) Fabrication process of anisotropic Janus microparticles compartmented with parachute-like (top row) and mushroom-like shapes (bottom row). (**b**) Optical microscope images of anisotropic Janus microparticles compartmented with parachute-like (left) and mushroom-like shapes (right). The inset is a sketch of Janus microparticle with anisotropic internal structures. Note that particles are moved into water chamber after UV curing to observe clearly and ascertain that the particle is cross-linked.

A simple fabrication method for the formation of anisotropic Janus microparticles with different internal shapes is proposed (Fig. 7). A positively charged droplet (purple) containing polyethylene (glycol) diacrylate (PEGDA) and SA, as well as a negatively charged droplet (colorless) containing PEGDA and CaCl_2_, are dispensed with the application of an almost equal and opposite voltage to each nozzle. Subsequently, the oppositely charged droplets are coalesced by electric attraction (Fig. 7a). After coalescence, the calcium alginate is formed at the interface between two droplets. Then, the merged droplet is polymerized by the irradiation of UV light source, which can produce anisotropic Janus microparticles with parachute- and mushroom-shaped internal structures. (Fig. 7b, Movie S1 in Supplementary Information). These Janus microparticles can be fabricated by changing the viscosity and surface tension of a droplet according to the PEGDA concentration^35^,^36^. To fabricate Janus microparticles with parachute-shaped internal structure, two droplets containing the same concentration of PEGDA are dispensed. However, a droplet containing PEGDA and SA has higher viscosity and lower surface tension than a droplet containing PEGDA and CaCl_2_ because of the characteristics of highly viscous SA solutions^37^. After coalescence of these droplets, surface tension gradient exhibited a tangential force along the interface of the merged droplet, which induced tangential flow (i.e., Marangoni convection) from the liquid with low surface tension (i.e., aqueous solution of PEGDA and SA) to that with a high surface tension (i.e., aqueous solution of PEGDA and CaCl_2_)^38^. However, the tangential flow is retarded by the viscous drag of SA solutions and the formation of calcium alginate. Consequently, Janus microparticles with parachute-shaped internal structure with a clearly defined interface are produced. To fabricate Janus microparticles with mushroom-shaped internal structure, two droplets containing different PEGDA concentrations are dispensed. The droplet containing PEGDA and CaCl_2_ has higher PEGDA concentration than that containing PEGDA and SA. Thus, the former has lower surface tension than the latter. This difference leads to tangential flow from the aqueous solution of PEGDA and CaCl_2_ to that of PEGDA and SA. Thus, the direction of this flow is opposite to that in case of Janus microparticles with parachute-shaped internal structure. Janus microparticles with mushroom-shaped internal structure with a clearly defined interface can be also produced by this flow and by calcium alginate. The shape of the Janus microparticles can be controlled by varying the physical properties of the droplet that contains UV-curable polymers, such as droplet volume, viscosity, and surface tension^39^,^40^,^41^. A systematic study on the shape variation of the Janus microparticles with these physical factors will be conducted in the future.

### Integration of ECC-induced droplet dispensing and electrophoretic manipulation system

**Figure 8:**
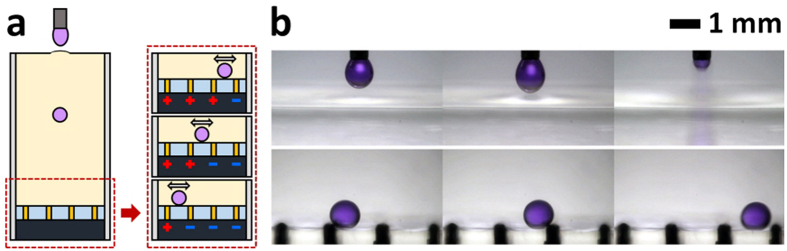
Combination of ECC-driven droplet dispensing and ECD-driven manipulation systems. (**a**) Conceptual description of the combined system. (**b**) Droplet dispensing (top row) and manipulating (bottom row) processes.

The proposed dispensing method can be easily integrated into digital microfluidic manipulation systems through electric control methods, such as electrowetting^42^,^43^,^44^, pre-charging method^45^,^46^, and electrophoresis of a charged droplet (ECD) method^47^,^48^,^49^. This ease of integration is facilitated by the generation of the charged droplets and the amount and polarity of charge on the droplet that can be tuned by the present dispensing method. In this study, the proposed dispensing system is integrated with an ECD-driven manipulation system, as illustrated in Fig. 8a. When a droplet submerged in oil medium comes into contact with an electrified electrode, the droplet acquires a charge. This charged droplet can then be manipulated by the electrophoretic force through a controllable electric field called the ECD method^47^,^48^.

To examine this idea, a droplet is preferentially dispensed into an oil chamber embedded with ECD-driven manipulation system by using the ECC-induced dispensing method (top row of Fig. 8b) to ascertain the feasibility of the proposed system. Notably, the dispensed droplet can be delivered toward the target (or preferred) position by controlling the polarity of the voltage exerted on the electrode because it is pre-charged by the ECC method. Subsequently, the droplet can be transported to the desired position by a controllable electric field (bottom row of Fig. 8b). The ECD-driven manipulation system presents several technological advantages, including minimum contact with solid surfaces, easy control by electric attraction, and manipulation of even solidified droplets^47^,^48^. Thus, the integration of the proposed dispensing method and electrically-controlled droplet manipulation system can provide more utility and flexibility in microfluidic applications related to synthesis of Janus particles^50^,^51^,^52^, three-dimensional (3D) assembly of lipid bilayers^53^, and 3D cell cultures^54^,^55^.

## Discussion

In this study, the dispensing of droplet into oil through the ECC method was systematically explored. The dispensing behavior was experimentally observed under a wide range of flow rates, applied voltages, and gap distances to determine the condition conducive for the generation of the charged droplet with uniform volume. Based on this dispensing platform, simple and straightforward methods to demonstrate the preparation of colloidosomes and the synthesis of microparticles were proposed. Finally, a combined system consisting of the ECC-induced droplet dispensing and ECD-driven manipulation systems was constructed.

Whereas the applicability of the proposed dispensing methods was successfully demonstrated by the aforementioned experiments, a notably high resolution is required by the ECC dispensing method for further practical applications. Based on the empirical relationship between droplet volume, voltage, flow rate and gap, the droplet volume in this study was mainly affected by applied voltage and gap distance. Because of the limitation in reduction of gap distance and bursting of dispensed droplets caused by excessive charge, it is not easy to dispense a droplet with uniform volume smaller than few tens of nanoliters by only tuning the applied voltage and gap in the present system. Consequently, the investigation of other parameters such as dynamic adhesion of interfaces^56^ and pulsed electric potential^57^,^58^ influencing on the volume of dispensed droplets will be conducted to determine the optimal conditions for a high-resolution ECC dispensing method in the near future.

## Methods

### Droplet dispensing system

The experimental setup employed in this study is similar to a typical apparatus used for typical EHD jetting systems. A transparent acrylic cell (6.7 cm length, 6.7 cm width, and 5 cm height) was filled with silicone oil (Shin-Etsu Silicone Korea Co., Ltd.) as non-conducting (or insulating) liquid with a kinematic viscosity of 100 cSt. A stainless steel nozzle (Hamilton, 20G; inner diameter = 0.6 mm, outer diameter = 0.9 mm) was used. The gap distance between the nozzle and the surface of silicone oil varied from 1 mm to 2 mm, with 0.5 mm increment. Distilled water (J. T. Baker®) as conducting liquid was provided using a syringe pump through the nozzle. The flow rate was changed from 5 μL·min^−1^ to 25 μL·min^−1^, with 5 μL·min^−1^ increment. A high DC voltage ranging from 0 kV to 4 kV, with 0.25 kV increment, was applied on the nozzle using a function generator (Agilent, 33220A) and a high-voltage power supply (Trek Co., 610E). The electric charges remaining on the oil chamber surface may cause an unexpected effect on the dispensing conditions of a charged droplet. To minimize the unwanted effects of the remaining charges, the charges were removed by gently rubbing with an earthed metal cylinder over the surface of the oil chamber prior to each experiment. The dispensing process of a charged droplet was recorded by a high-speed camera (Photron, Fastcam-1024 PCI) integrated with a zoom lens (Edmund Optics, VZMTM 450i eo) at 500 fps. Digital image processing and data analysis were performed with LabVIEW software (National Instruments, Austin, TX). Each experiment was repeated at least three times; all data presented were measurement means.

### Preparation of colloidosomes

To prepare monodispersed colloidosomes containing particles with different sizes, two types of PS particles were dispersed into 20 mL of n-dodecane oil (Alfa Aesar, 99% purity): (I) PS particles (Sigma Aldrich, 10% solid in 5 mL, diameter = 10 μm) and (II) fluorescent PS-COOH particles (Sigma Aldrich, 2.5% solid in 1 mL, average diameter = 2 μm, λ_ex_/λ_em_ = 575 nm/610 nm). Originally, these particles were received as aqueous suspensions with small amount of surfactant. To remove suspension residues, such as water and surfactant from particles, the aqueous suspension was washed, centrifuged several times, and then dried in vacuum oven at 60 °C overnight. The dehydrated particles were re-dispersed into n-dodecane oil using ultrasonic bath. The particle encapsulation process of a charged droplet was recorded consecutively with a high-speed camera. A layered film of fluorescent PS-COOH particles formed on a charged droplet was observed by a confocal laser scanning microscope (Leica Microsystems Ltd., TCS SP5 II).

### Chemical reactions and synthesis of Janus particles

Two nozzles and high-voltage power suppliers (Trek Co., 677B) were used to merge oppositely charged droplets. The distance between the two nozzles was 2 cm. A transparent acrylic cell (6.7 cm length, 6.7 cm width, and 5 cm height) was filled with silicone oil with a kinematic viscosity of 20 cSt (Shin-Etsu Silicone Korea Co., Ltd.). The Na_2_CO_3_ solution (0.29 M, Samchun Chemical Co.) and the CaCl_2_ solution (0.27 M, Junsei Chemical Co.) with 1 mM phenol red were supplied through the two nozzles at same flow rate of 53 μL·min^−1^ to conduct experiments of chemical reaction for precipitate formation. An equal and opposite voltage (±1 kV) was applied to each nozzle. The distance between the nozzles and the surface of the silicone oil was fixed at 1 mm. The CaCl_2_ solution (0.2 M) and SA solution (1 wt%, Samchun Chemical Co.) mixed with 0.2 mM phenol red were supplied through the two nozzles at flow rate of 15 and 20 μL·min^−1^, respectively, to form the Janus droplet. Ethanol (14 wt%, J.T. Baker) was then added to decrease the surface tension of CaCl_2_ solution. SA, CaCl_2_, PEGDA (M_w_ = 575 g·mol^−1^, Sigma Aldrich), and 2-hydroxy-1-[4-(hydroxyethoxy) phenyl]-2-methyl-1-propanone (Irgacure 2959, Ciba Specialty Chemicals) were prepared to demonstrate the fabrication of Janus microparticles. PEGDA and Irgacure 2959 were used as crosslinking material and photo initiator, respectively. Three solutions were prepared to produce Janus microparticles with different shapes: (I) mixture of SA solution (0.5 wt%) and PEGDA (30 wt%); (II) mixture of CaCl_2_ (0.05 M) and PEGDA (30 wt%); and (III) mixture of CaCl_2_ (0.05 M) and PEGDA (50 wt%). All solutions contained irgacure 2959 (1.5 wt%). A UV light source (Lightingcure^TM^, Hamamatsu, LC8) was irradiated for polymerization of merged droplets. The coalescence process of oppositely charged droplets was recorded by a digital camera (Canon, EOS 1100D) integrated with a macro lens (canon, MP-E 65 mm). The optical images of the Janus microparticles were acquired by microscope (Nikon, Eclipse Ti-S).

### Combination of droplet dispensing and manipulation systems

The ECD method was employed to manipulate a charged droplet dispensed by the ECC method. This study briefly explains the experimental setup for ECD method; additional details can be found in Im *et al.*’s^47^ paper. The array of electrodes used for the ECD system was created through the assembly of conventional pin header sockets with 2.54 mm pitch. The electrode array was combined with a transparent acrylic cell (2 cm length, 1.8 cm width, and 3.5 cm height). The cell was filled with silicone oil having a kinematic viscosity of 6 cSt (Dow Corning, DC200F). The polarity of each electrode control was independently controlled by relay (LG, RY 5W-K), LabVIEW software, and a high-voltage power source (Ultravolt, 0.2US5-P0.1, 0.2US5-N0.1).

## Additional Information

**How to cite this article**: Um, T. *et al.* Electrically Controllable Microparticle Synthesis and Digital Microfluidic Manipulation by Electric-Field-Induced Droplet Dispensing into Immiscible Fluids. *Sci. Rep.* **6**, 31901; doi: 10.1038/srep31901 (2016).

## Supplementary Material

Supplementary Information

Supplementary Movie

## Figures and Tables

**Figure 1:**
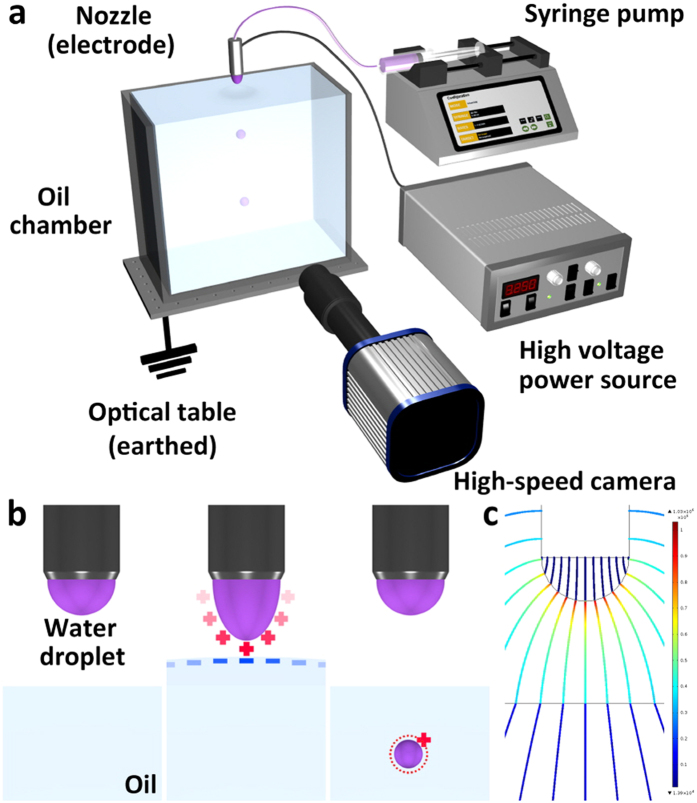
(**a**) Schematic of the experimental setup used for ECC–induced droplet dispensing into oil. The oil chamber is positioned on the optical table, which is earthed and thus acts as a ground electrode. (**b**) Schematic diagrams of ECC–induced dispensing process. (**c**) Spatial distribution of electric field in ECC-induced dispensing. The detailed information about simulation is available on Figure S1 in Supplementary Information.
