# Supplementary material for: Electrically Controllable Microparticle Synthesis and Digital Microfluidic Manipulation by Electric-Field-Induced Droplet Dispensing into Immiscible Fluids
Source: Sci Rep. 2016 Aug 18;6:31901. doi: 10.1038/srep31901 (PMC4989170; doi:10.1038/srep31901)
Supplement: Supplementary Information [file srep31901-s1.doc]

**Supplementary Information**

**Electrically Controllable Microparticle Synthesis and Digital Microfluidic Manipulation by Electric-Field-Induced Droplet Dispensing into Immiscible Fluids**

Taewoong Um^†1^, Jiwoo Hong^†2^, Do Jin Im^3^, Sang Joon Lee^2*^, and In Seok Kang^1*^

^1^Department of Chemical Engineering, Pohang University of Science and Technology (POSTECH), San 31, Hyoja-Dong, Nam-Gu, Pohang, Gyeongbuk, 37673, South Korea.

^2^Department of Mechanical Engineering, Pohang University of Science and Technology (POSTECH), San 31, Hyoja-Dong, Nam-Gu, Pohang, Gyeongbuk, 37673, South Korea.

^3^Department of Chemical Engineering, Pukyong National University, 365 Sinseon-ro, Nam-gu, Busan 48547, South Korea.

^*^Corresponding author.

Tel.: +82-54-279-2273. Fax: +82-54-279-2699. E-mail: iskang@postech.ac.kr.

Tel.: +82-54-279-2169. Fax: +82-54-279-3199. E-mail: sjlee@postech.ac.kr.

†These authors equally contributed to this work.

**Supplementary Figures**

**1. The 2D numerical simulation of ECC-induced dispensing method.**

We conduct 2D numerical simulation to calculate the distribution of electric field and polarization in ECC-induced dispensing. In the numerical model, an electric potential (+2.5 kV) and ground conditions are imposed to the surface of the nozzle and the bottom surface of the oil chamber as boundary conditions, respectively. The charge conservation condition is applied on the all domains with governing equations $\mathbf{E}=-\nabla V$and $\nabla\cdot\mathbf{D}=\rho_{v}$. The electric displacement $\mathbf{D}$ is equal to $\varepsilon_{0}\varepsilon_{r}\mathbf{E}$, where $\varepsilon_{0}$ is permittivity of free space, $\varepsilon_{r}$ is relative permittivity and $\mathbf{E}$ is an electric field. The zero charge condition ($\mathbf{n}\cdot\mathbf{D}=0$, where $\mathbf{n}$ is unit normal vector) is applied at the outermost boundaries. The relative permittivities of air, water, silicone oil, and Poly (methyl methacrylate) (PMMA) chamber are used as 1, 78.5, 2.8^S1^ and 3.6^S2^.


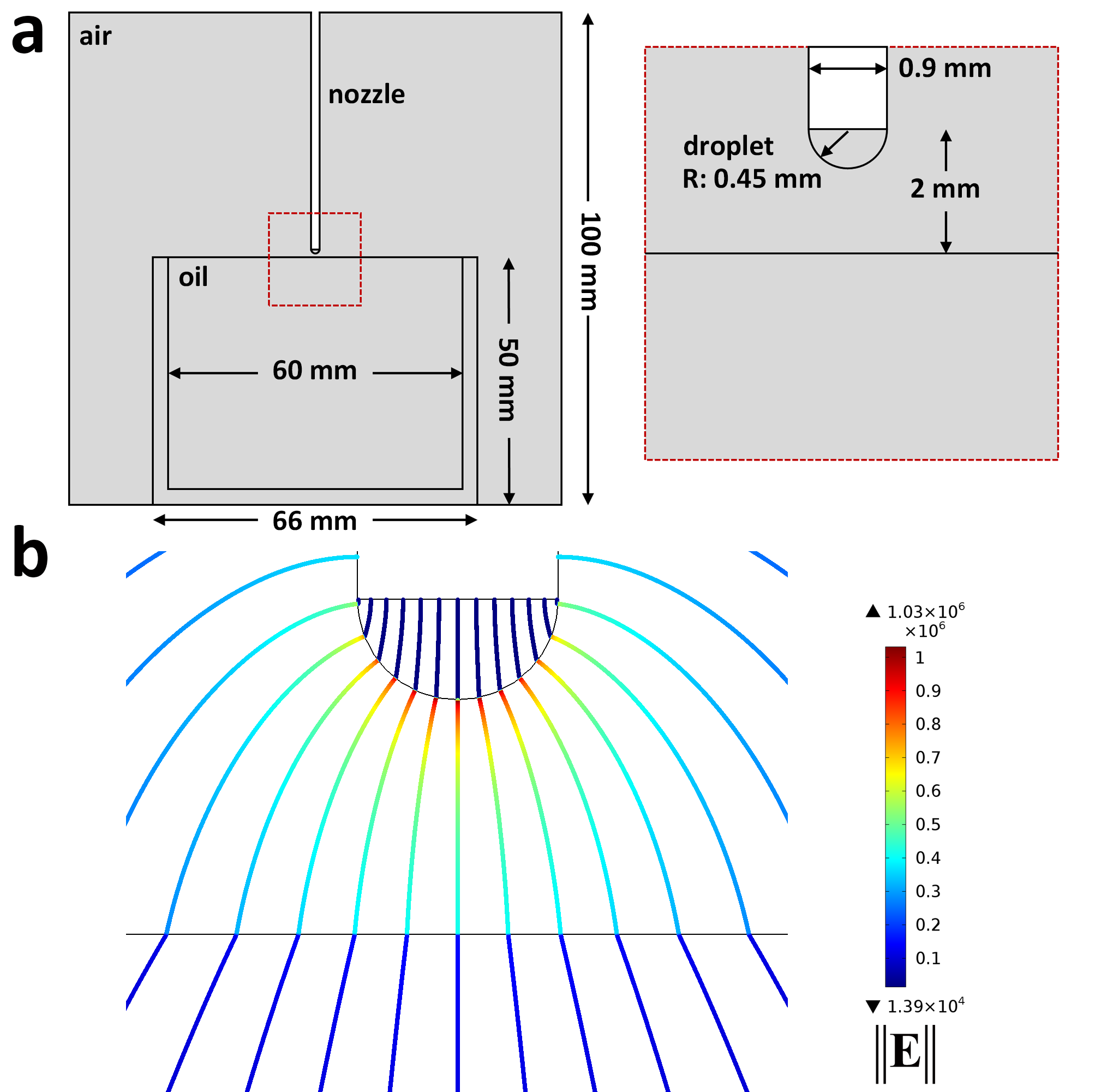


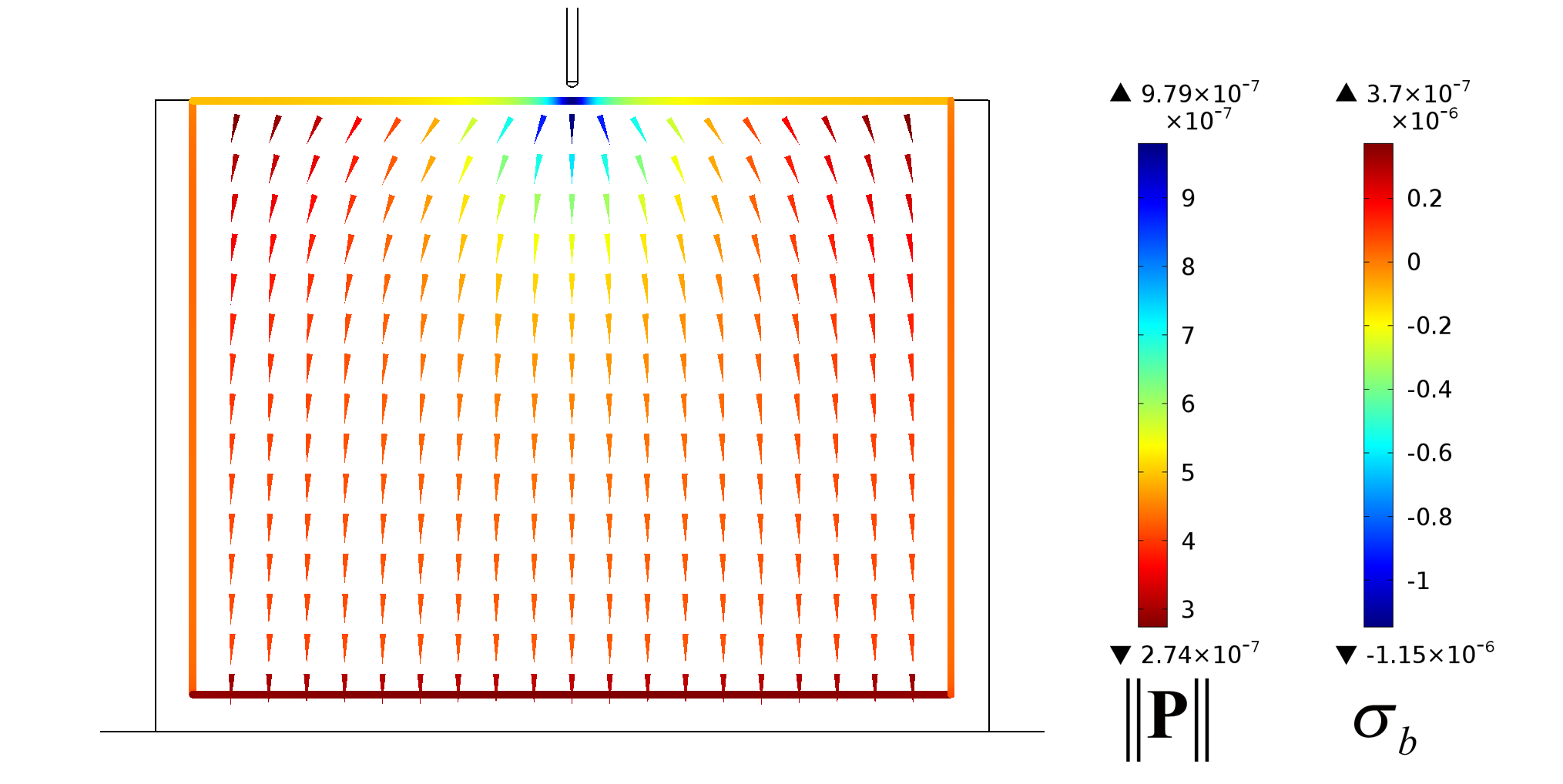


**Figure S1.** The 2D numerical simulation of ECC-induced dispensing. (a) The computational domain of the numerical simulation. (b) The electric field distribution between the droplet and the oil interface. (c) The distributions of polarization $\mathbf{P}$ and surface bound charge density $\sigma_{b}$ of oil medium. Here, arrows and legends denote the direction and magnitude of polarization, respectively. The colored line at the oil interface indicates spatial distribution of the surface bound charge density [C/m^2^].

**References**

S1. Israelachvili, J. N. *Intermolecular and surface forces 2nd edn*. Ch. 3, 41 (Academic press, 1991)

S2. Hsu, S. L. Poly(methyl methacrylate) in *Polymer data handbook* (ed. James, E. M.) 656 (Oxford University Press, 1999)

**2. The charge amount on the droplet dispensed by ECC method**

To measure amount of electric charge on droplets dispensed by the ECC method, droplet trajectory method was used.^S1^ The amount of electric charge can be estimated by this method from the trajectory of a droplet dispensed in oil. After a droplet is dispensed into oil by the ECC method, a uniform electric field is applied between the two parallel electrodes. The electric field is generated by using a voltage supplier (Trek Model 677B). The pathway of the droplet is consecutively recorded by using a high speed camera (Photron, Fastcam-1024 PCI) and subsequently velocity of the droplet is calculated from acquired images by digital image processing with Labview^TM^. Consequently, the charge of the droplet is estimated from force balance between the electrical and drag forces.^S2^ Because of additional electric field between nozzle and electrodes in the chamber, the bursting of droplet appears at smaller voltage (2.75 kV) than in the case of size measurement experiment. As illustrated in Figure S2, the charge amount of droplet increases toward Rayleigh’s charge limit as an applied voltage increases.

**
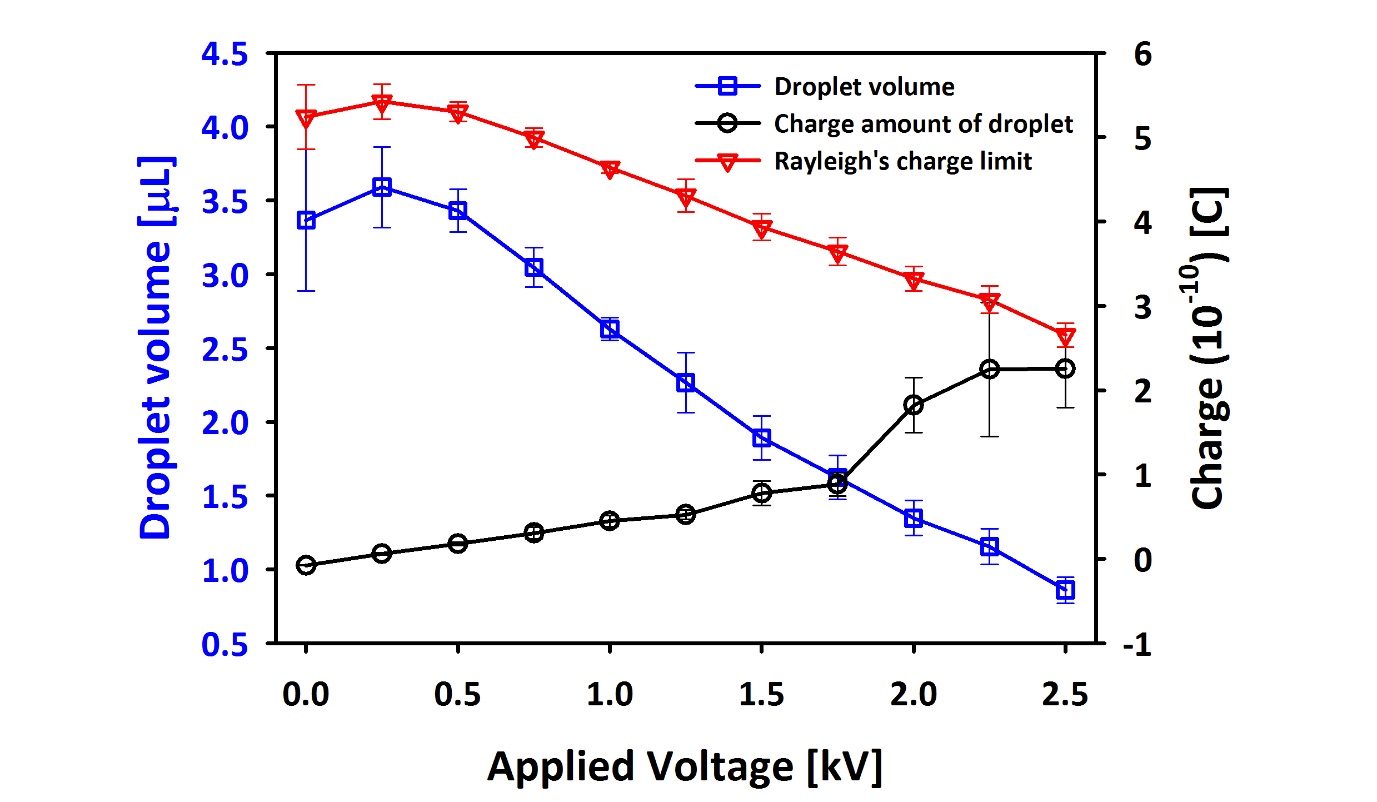
**

**Figure S2**. Electric charge amount on dispensed droplets with respect to applied voltage. (flow rate = 15 μL/min, gap = 2 mm). As applied voltage increases, the charge amounts of droplet also increase toward Rayleigh’s charge limit.

**References**

S1. Choi D. *et al.* Spontaneous electrical charging of droplets by conventional pipetting. *Sci. Rep.* **3**, 2037 (2013).

S2. Leal, L. G. *Advanced Transport Phenomena: Fluid Mechanics and Convective Transport Processes*. (Cambridge University Press, 2007).

**3. The packing condition of colloidosomes generated by ECC method.**

The packing condition of the colloidosomes is observed by microscope and confocal fluorescent microscope. The aggregated particles are initially observed by microscope, and the surface of droplet is carefully checked by the microscope to observe the surface of the colloidosome. As shown in Figure S3(c), the fluorescent intensity becomes high due to aggregation of particles.


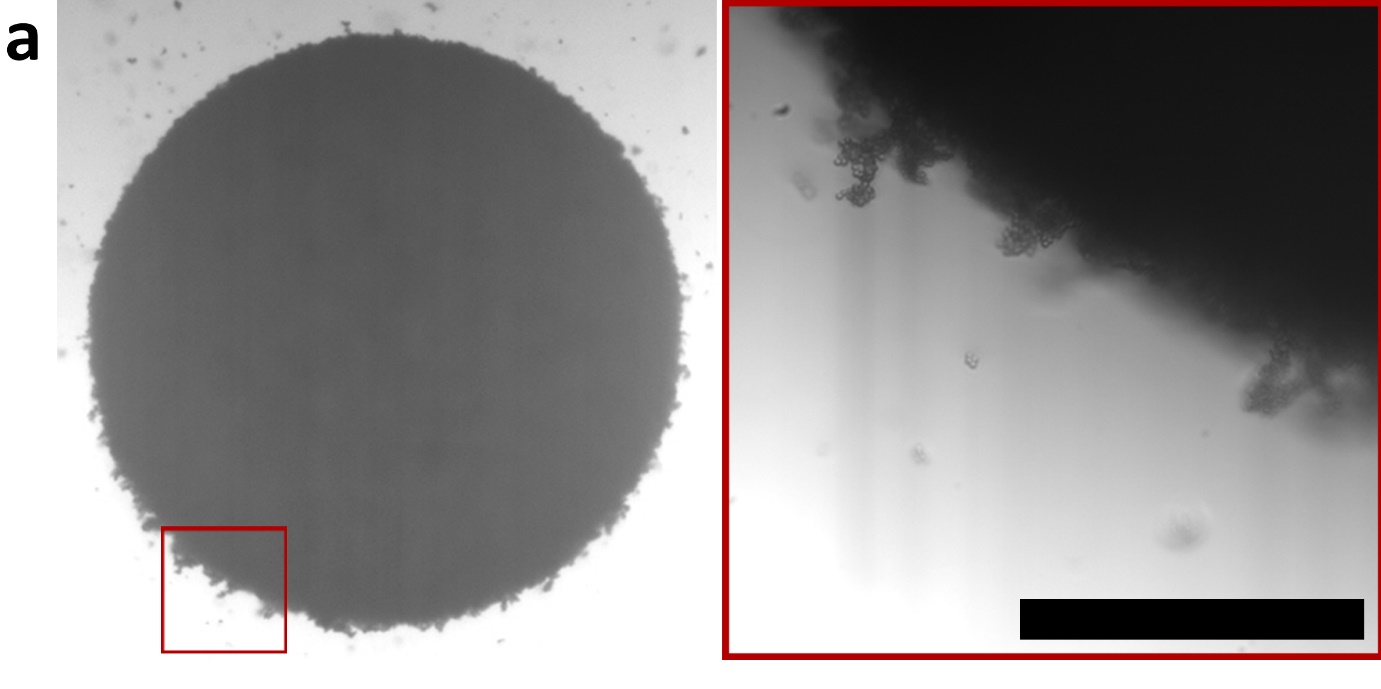

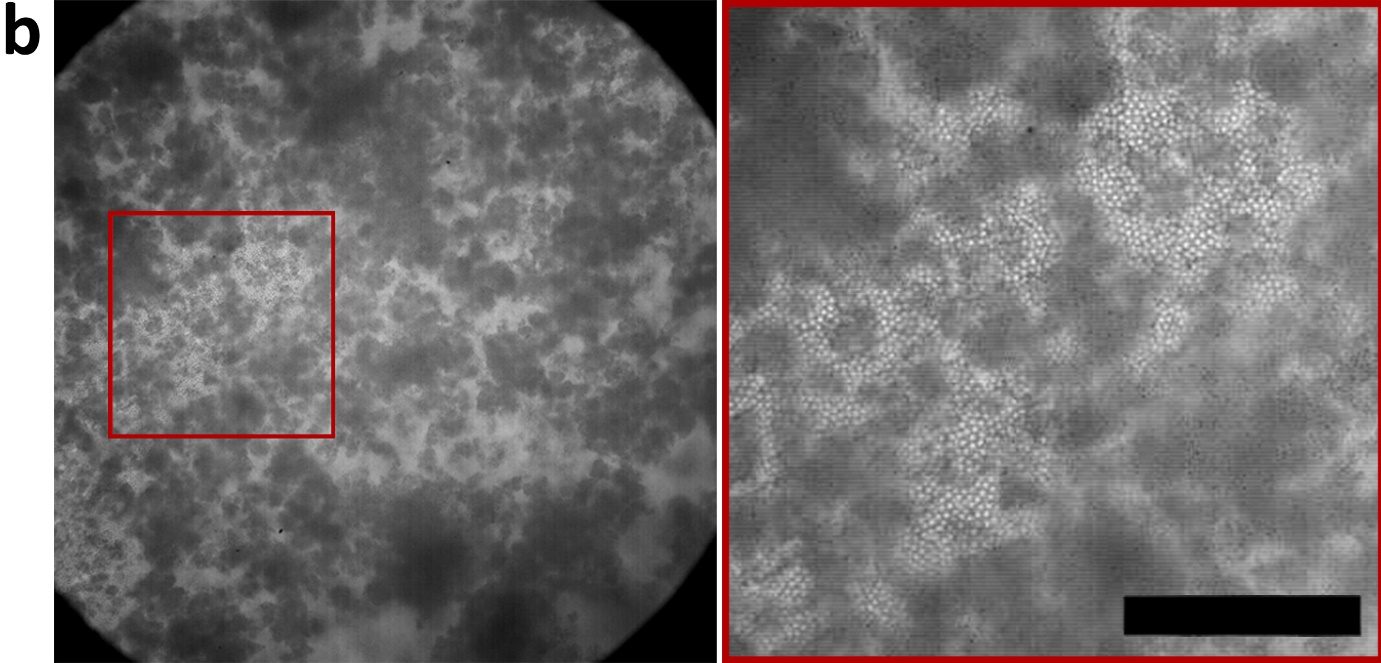

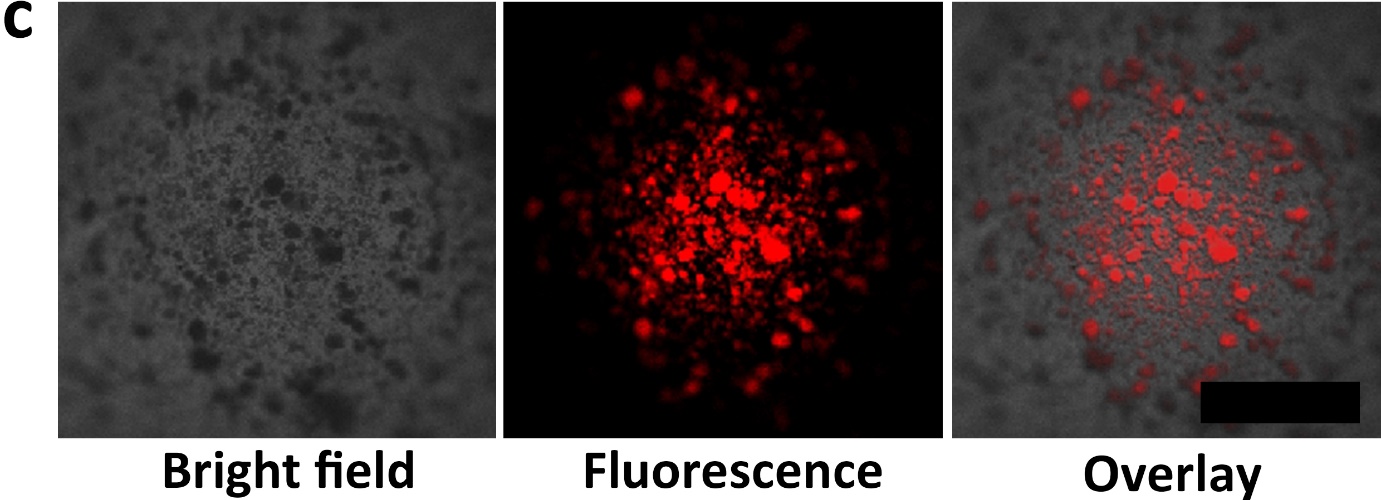


**Figure S3. microscope images of the droplet packed by particles.** (a) The aggregated particles on the surface of droplet. Scale bar is 50 μm. (b) The surface of droplet packed by particles. Scale bar is 50 μm. (c) The bright field microscope and confocal microscope images of the droplet. Scale bar is 150 μm.

**4. The behaviors of carboxylate-modified polystyrene particles (PS-COOH) under different potentials.**

To confirm the behaviors of PS-COOH under positive and negative potential, pendant droplet on the nozzle is dipped into dodecane oil. Then the electric potential is applied on the nozzle at 2 s. Applying voltage higher than 2 kV could result in the breakup of droplet, relatively small (1.5 kV) voltage is applied to observe the behaviors of the particles. Then, the confocal microscope is used to check that the overall surface of droplet is covered by particles.


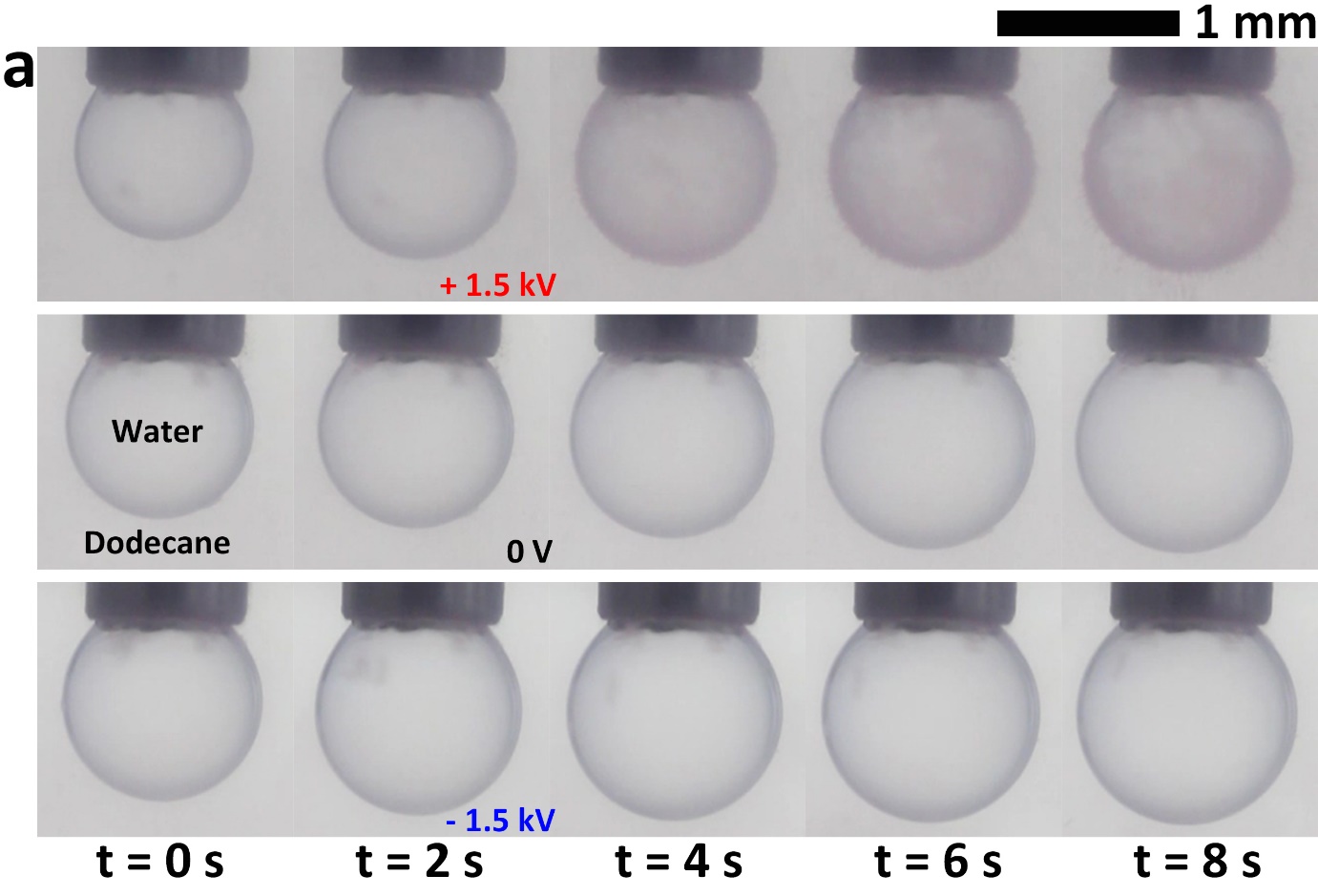

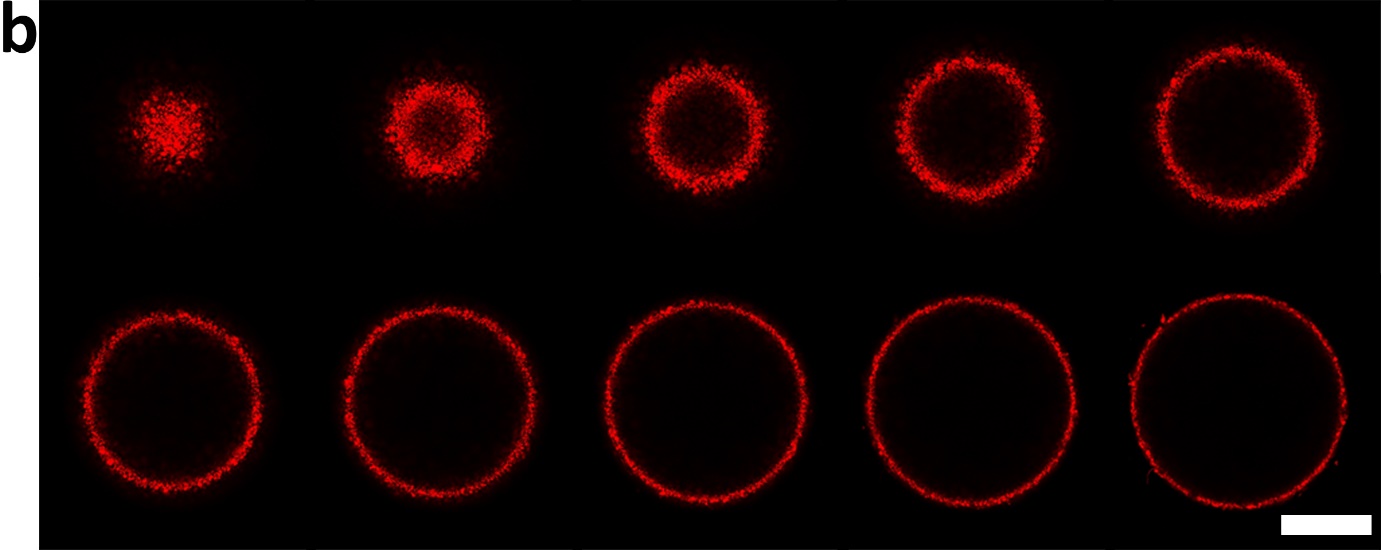


**Figure S4.** (a) The behaviors of PS-COOH particles under positive (1.5 kV), zero, negative (1.5 kV) voltages. The nozzle is dipped into dodecane oil where PS-COOH particles are dispersed (0.0625% solids in 1mL). When negative voltage is applied, the fluctuation of PS-COOH particles is appeared, but it doesn’t lead to adsorption of particles onto interface. (b) The droplet covered by particles is shown by series of confocal z-slices. Scale bar is 500 μm.

**5. The effects of reversed electrical polarity to the morphology of anisotropic Janus particle.**

At first, (1) mixture of SA solution (0.5 wt%) and PEGDA (30 wt%) and (2) mixture of CaCl_2_ (0.05 M) and PEGDA (50 wt%) are used to make mushroom-like-shaped Janus microparticle same as in the manuscript (Figure S5(a), positive potential on SA solution, negative potential on CaCl_2_ solution). Although the polarity is reversed, the mushroom-like-shaped Janus microparticle same as in previous case is formed (Figure S5(b)).


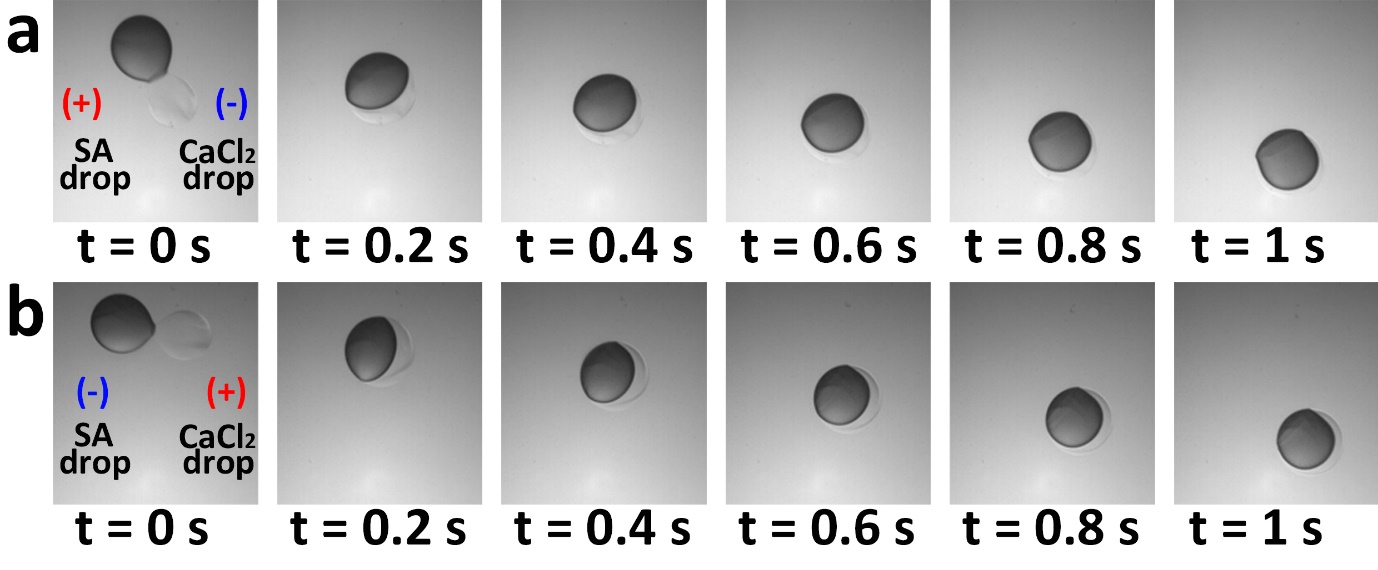


**Figure S5.** The time lapse images of the morphology formation after the coalescence of SA containing droplet (dark) and CaCl_2_ droplet (transparent). (a) Negative potential on the SA containing droplet and positive potential on the CaCl_2_ droplet. (b) Positive potential on the SA containing droplet and negative potential on the CaCl_2_ droplet. The morphologies of the Janus particle become mushroom shaped particle in both (a) and (b) cases.

**Supplementary Table**

**1. The surface tensions and viscosities of the solutions.**

The surface tensions of all the solutions are measured by pendant droplet tensiometry^S1^ at 25. 5 ºC. The viscosities of all the solutions are measured by a viscometer (LV DV-II+ Pro Brookfield Programmable Viscometer, USA) with spindle (SC4-18, viscosities range: 1,5 – 30,000 cP) at 23.5 ºC.

**Table S1. The surface tensions and viscosities of the solutions.**

| **Materials** | **Surface tension (mN/m)** | **Viscosity (cP)** |
| --- | --- | --- |
| Water | 71.9464±0.3279 | 1.06±0.2 |
| Water + Ethanol (1.44) with 0.2 M CaCl_2_ | 43.4079±0.3605 | 1.78±0.2 |
| 0.2 M CaCl_2_ solution | 73.8572±0.6145 | 1.26±0.2 |
| Sodium alginate (1) solution | 68.3108±0.3341 | 42.5±0.6 |
| PEGDA (30) +SA (0.5)+ Irgacure 2959 (1.5) solution | 41.2310±0.1186 | 26.1±0.375 |
| PEGDA (30) +0.05 M CaCl_2_+Irgacure 2959 (1.5) solution | 41.4188±0.1795 | 3.52±0.2 |
| PEGDA (50) +0.05 M CaCl_2_+Irgacure 2959 (1.5) solution | 40.9680±0.2394 | 8.72±0.2 |

*The surface tensions of all the solution is measured by 3 independent sets.

*the number in parentheses means wt%

**Reference**

S1. Berry, J. D., Neeson, M. J., Dagastine, R. R., Chan, D. Y. C., Tabor, R. F. Measurement of

surface and interfacial tension using pendant drop tensiometry. *J. Colloid Interface Sci.* **454**, 226-237 (2015).
